# Frailty Risk After a Cardiovascular Event Among Community-Dwelling Older People: Influence of Sociodemographic, Polypharmacy, and Pre-event Frailty

**DOI:** 10.1016/j.cjca.2025.06.003

**Published:** 2025-06-11

**Authors:** Aung Zaw Zaw Phyo, Andrew Tonkin, Sara E. Espinoza, A.R.M. Saifuddin Ekram, Robyn L. Woods, Joanne Ryan

**Affiliations:** a School of Public Health and Preventive Medicine, Monash University, Melbourne, Victoria, Australia; b Center for Translational Geroscience, Department of Medicine, Cedars-Sinai Medical Center, Los Angeles, California, USA

**Keywords:** frailty risk, cardiovascular disease, community-dwelling older people, sociodemographic, polypharmacy

## Abstract

**Background:**

Frailty is a significant concern for older adults and can increase after a major health event. This study examined the risk of incident frailty after a cardiovascular disease (CVD) event among community-dwelling older people aged ≥ 65 years and explored whether sociodemographic factors, polypharmacy, and pre-event frailty influence their risk of developing frailty after a CVD event.

**Methods:**

This study included a cohort of 738 participants (38.5% women) from the ASPREE study who were not classified as frail before their CVD event. Frailty was measured annually using the 64-item deficit-accumulation frailty index.

**Results:**

Over an average 2.6 years after a CVD event, 333 individuals had incident frailty. In logistic regression models, increased chronological age, being a woman, and having polypharmacy were associated with 4% to 83% increased odds of developing frailty after a CVD event. Individuals with CVD residing in the inner regional area had about 50% higher odds of having frailty than those living in cities. This association was more evident among stroke survivors, with both inner regional (adjusted odds ratio [OR] 2.13) and outer regional or remote residents (adjusted OR 2.37) having greater odds of frailty. Individuals who were classified as pre-frail before their CVD event, had notably higher odds of progressing to frailty after the CVD event (adjusted OR 3.41).

**Conclusions:**

Our community-based study provides robust evidence that individuals who were women, older, pre-frail, with polypharmacy, and living in regional or remote areas have a markedly greater odds of developing frailty after a CVD event.

## RÉSUMÉ

**Contexte:**

La fragilité est une préoccupation importante chez les personnes âgées et peut s’intensifier après un problème de santé majeur. Cette étude a été menée pour examiner a) le risque de fragilité d’apparition nouvelle après un événement cardiovasculaire (CV) chez des personnes âgées (≥ 65 ans) vivant dans la communauté et b) si les facteurs sociodémographiques, la polypharmacie et la fragilité avant l’événement influencent le risque de fragilité après l’événement.

**Méthodologie:**

Cette étude a été réalisée dans une cohorte de 738 personnes (38,5 % de femmes) ayant participé à l’étude ASPREE qui n’étaient pas considérées comme fragiles avant l’événement CV. La fragilité a été évaluée chaque année à l’aide de l’indice de fragilité en fonction des déficits accumulés à 64 items.

**Résultats:**

En l’espace de 2,6 ans après un événement CV en moyenne, 333 personnes ont présenté une fragilité d’apparition nouvelle. Dans les modèles de régression logistique, l’augmentation de l’âge chronologique, le sexe féminin et la polypharmacie ont été associés à une augmentation de 4 à 83 % du risque de fragilité après un événement CV. Le risque de fragilité était environ 50 % plus élevé chez les personnes atteintes d’une maladie CV résidant dans une zone semi-urbaine que chez celles résidant en zone urbaine. Ce lien était le plus évident chez les survivants d’un accident vasculaire cérébral (AVC), le risque de fragilité le plus important étant observé chez les personnes résidant en zone semi-urbaine (rapport de cotes [RC] ajusté : 2,13) et chez les personnes résidant en zone isolée/éloignée (RC ajusté : 2,37). Les personnes déjà considéreés fragiles avant l’événement CV présentaient un risque nettement plus élevé de fragilité après l’événement CV (RC ajusté : 3,41).

**Conclusions:**

Notre étude dans la communauté indique clairement que le sexe féminin, l’âge avancé parallèlement à une fragilité preéxistante, la polypharmacie et le fait de résider dans une région isolée/éloignée augmentent considérablement le risque de présenter une fragilité après un événement CV.

Frailty is a common clinical syndrome among older people.^1^ It is characterised by physiologic function and reserve deteriorations across multiple organ systems, resulting in heightened vulnerability and reduced resilience to illnesses and stressors.^1,2^ Cardiovascular disease (CVD) is one of the leading causes of morbidity and mortality worldwide and a major cause of long-term disability in older people.^3^ The relationship between frailty and CVD is bidirectional. Previous research, including our study, has shown that frailty measured at 1 point in time is an effective indicator of CVD risk.^4,5^ In addition, evidence has been shown that frail people who have had CVD events are at a higher risk of adverse reactions to medications or surgical procedures, and are more likely to have prolonged recovery times, higher rates of readmission, and a greater need for long-term residential care.^6,7^ As such, frailty is increasingly acknowledged as a syndrome that influences treatment outcomes and overall prognosis for those with a CVD event, underscoring the importance of integrating frailty risk identification and management into CVD treatment.^8-10^

There are some studies examining frailty risk among CVD patients in hospital settings, particularly among people with heart failure, with a transplanted heart, or after cardiac surgery, that can inform understanding of the impact of frailty on clinical outcomes.^11-14^ Yet, there remains limited knowledge about frailty among community-dwelling people after a CVD event. Community-dwelling individuals with a CVD event may experience challenges in accessing health care and self-managing their health conditions owing to their area of residence, living status, and socioeconomic status (SES).^15-19^ Thus, understanding the post-CVD frailty burden and its determinants in the community context is critical to facilitate early detection and the initiation of targeted interventions for community-dwelling people at most risk of frailty following a CVD event. This approach ultimately can help prevent the onset of post-CVD frailty and mitigate its progression at the primary care level, thereby improving the long-term prognosis for these individuals.

Therefore, this study aimed to estimate the incidence of frailty following a CVD event among community-dwelling older people and to examine whether chronological age, gender, living status, area of residence, SES, polypharmacy, and pre-event frailty status are associated with the risk of developing frailty after a CVD event.

## Methods

### Study population

This study used longitudinal data from the Aspirin in Reducing Events in the Elderly (ASPREE; NCT01038583) study.^20^ ASPREE was the first primary prevention clinical trial conducted to test the effectiveness of low-dose aspirin in promoting healthy aging among older individuals.^20^ From March 2010 to December 2014, ASPREE recruited 19,114 initially healthy individuals (87% Australian) aged ≥ 70 years (or ≥ 65 years for US minorities) who had no previous overt CVD events or other known 5-year life-limiting conditions.^20,21^ Further details of the ASPREE study design and inclusion criteria have been presented previously.^20,21^ The treatment phase of the ASPREE trial ended on June 12, 2017, and the Aspirin in Reducing Events in the Elderly—Extension (ASPREE-XT) observational study continues to follow these participants with the collection of extensive clinical measures and phenotypic data.^22^

The present study included 738 ASPREE participants (90.4% Australian) who had experienced a nonfatal CVD event and were not classified as frail before their CVD event. CVD was a prespecified composite secondary end point of ASPREE, consisting of nonfatal myocardial infarction (MI), nonfatal stroke, coronary heart disease, and hospitalisation for heart failure (HHF).^23^ All incident CVD events were adjudicated by expert clinical panels from Australia and the US.^23^

The ASPREE study complied with the Declaration of Helsinki and was approved by multiple institutional review boards in the US and Australia (www.aspree.org). The present study was approved by the Monash University Human Research Ethics Committee (MUHREC 42957).

### Frailty assessment and incident frailty

Frailty was annually assessed by means of the deficit-accumulation frailty index (FI).^24^ The FI was developed based on yearly protocol-driven measures captured in ASPREE, following the method of Rockwood et al.^24,25^ The original FI of the ASPREE study consisted of 67 items, and the detailed calculation was been reported previously.^25^ In the present study, we excluded the 3 items related to the CVD diagnoses (ie, stroke, MI, and HHF) from the FI calculation to capture the true effect of the CVD event on frailty.^26^ Thus, the FI used in this study consists of 64 items covering 8 disease diagnoses including cancer, osteoarthritis, and osteoporosis, 13 disease indicators including smoking, central adiposity, and self-rated health status, 26 functional deficits related to completing the activities of daily living, 11 mental and psychosocial deficits, 4 cognitive function measures, and 2 physical performance measures (grip strength and gait speed).^25,26^ Each item was scored ranging from 0 (deficit absent) to 1 (deficit present). The FI was calculated when at least 50 items were available and was estimated as the number of deficits noted for each person divided by the total number of possible deficits. The total score ranged from 0 to 1. Individuals were classified as nonfrail (≤ 0.10), pre-frail (> 0.10 and ≤ 0.21), or frail (> 0.21).^25^

### Other factors investigated

Based on previous literature^15-18^ and the World Health Organisation’s social determinants conceptual framework,^19^ the factors investigated included age, sex (male or female), living status (living alone or with family/others), area of residence (major cities, inner regional, or outer regional/remote), socioeconomic status SES (low, middle, or high), frailty status before a CVD event (nonfrail or pre-frail), and polypharmacy (< 5 medications or ≥ 5 medications).^27^ Data on these factors were obtained from the closest follow-up before a CVD event. Of note, residential postcode information was available only for Australian participants, enabling the estimation of the area of residence and SES within our Australian cohort. SES was estimated using the Socio-Economic Indexes for Areas—Index of Relative Socioeconomic Advantage and Disadvantage, based on information from the 2011 Australian Census.^28^ Regarding polypharmacy, the details have been published previously.^27^ In brief, polypharmacy was estimated using the data collected on prescription medications, which was categorised into no polypharmacy (< 5 medications) and polypharmacy (≥ 5 medications).^27^ This definition did not include food supplements, homeopathic medicine, or over-the-counter nonprescription medications.

### Statistical analyses

All analyses were undertaken using Stata software, release 17 (StataCorp). Participants’ characteristics were described using mean ± SD or n (%) as appropriate. Logistic regression models were used to examine the associations between our investigated factors (ie, age, sex, living status, area of residence, SES, frailty status before a CVD event, and polypharmacy) and the odds of incident FI-defined frailty after a CVD event. To minimise the potential bias introduced by overadjustments for variables, commonly known as the Table 2 fallacy,^29^ models were adjusted for age and sex only. The model was run separately for each factor under investigation.

Furthermore, analyses were repeated to examine the associations between these factors and the odds of incident frailty after the specific CVD subtypes available in ASPREE (ie, an MI or stroke event). Of note, in the ASPREE study, MI was identified based on the joint guidelines of the European Society of Cardiology and the American College of Cardiology.^30^ Nonfatal stroke was identified according to the World Health Organisation’s definition of rapidly developing clinical signs of focal or global disturbance of cerebral function lasting more than 24 hours (unless interrupted by surgery or death), with no apparent cause other than ischemic or hemorrhagic cerebrovascular disease.^31^

To assess the robustness of our findings, the analysis was repeated by excluding participants who experienced a second CVD event during the study period after their initial event. In addition, sensitivity analyses were conducted by excluding participants who were already pre-frail before experiencing a CVD event. To see multiple adjusted effect estimates from a single model, additional sensitivity analyses were conducted by running the model with the inclusion of all investigated factors and potential covariates: ethnoracial group, smoking status, hypertension, diabetes, dyslipidemia, obesity, depression, Modified Mini-Mental State Examination (3MS) score,^32^ and estimated glomerular filtration rate (eGFR) based on the Chronic Kidney Disease—Epidemiology Collaboration collaboration equation.^33^

### Additional analyses

To investigate the robustness of these findings using an alternative frailty assessment, analyses were repeated with a cohort of 651 individuals who had data for the Fried frailty phenotype. A modified Fried frailty phenotype was defined according to the following 5 core criteria: shrinking, weakness, exhaustion, slowness, and low activity.^1,34^ Details of this modified Fried phenotype have been reported previously.^5,34^ Each criterion was scored 1 for its presence or 0 for its absence. A total score ranges from 0 to 5. Individuals were classified as nonfrail (a score of 0), pre-frail (a score of 1-2), or frail (a score of 3-5).^5,34^

## Results

This study included 738 individuals who experienced a CVD event (38.5% women), none of whom were classified as frail before their CVD event. The study participants’ mean chronological age was 79.3 ± 5.0 years (Table 1). Most participants were white Australians (88.8%). Nearly half of the participants (46.3%) were taking ≥ 5 medications at the time of their CVD event.

Over an average follow-up of 2.6 years after a CVD event, 333 of 738 individuals (45.12%) were identified as having incident frailty according to FI (Central Illustration). Table 2 presents the age- and sex-adjusted associations between our investigated factors and the odds of developing frailty after a CVD event. A 1-year increase in chronological age was associated with a 4% rise in the odds of incident frailty after a CVD event. Females, compared with males, had 83% higher odds of developing post-CVD frailty. In terms of the area of residence, individuals from the inner regional area had approximately 50% increased odds of having frailty after a CVD event compared with those living in cities. This significant association was also observed among stroke survivors, with both inner regional (odds ratio [OR] 2.13) and outer regional and remote residents (OR 2.37) experiencing greater odds of incident frailty than those living in major cities (Table 3). Having polypharmacy (ie, ≥ 5 medications) was associated with 37% increased odds of developing incident frailty after a CVD event (Table 2). There was no evidence of the associations between polypharmacy and incident frailty among MI and stroke survivors (Table 3).

Pre-event frailty status was noticeably associated with increased odds of developing incident frailty after a CVD event, with this association consistently found across the CVD subtypes of MI and stroke (Tables 2 and 3). Specifically, compared with individuals who were not frail before an event, those who were classified as pre-frail beforehand had markedly higher odds of developing into frailty following a CVD event (OR 3.41), an MI event (OR 4.45), or stroke (OR 3.40).

Similar findings were observed in a sensitivity analysis that excluded participants who experienced a second CVD event during the study period after their initial event (Supplemental Table S1). When undertaking sensitivity analysis with the inclusion of all investigated factors and potential covariates in a single model, most findings were largely consistent (Supplemental Tables S2, S3, and S4). There was no evidence of an association between polypharmacy and incident frailty (Supplemental Table S2). The sex difference in experiencing post-CVD frailty remained significant even after additionally excluding individuals who were already pre-frail before their CVD event (Supplemental Table S5); females had markedly higher odds of developing into frailty after a CVD event (OR 2.16) or stroke (OR 2.09) compared with males.

In additional analyses, over an average of 3 years’ follow-up after a CVD event, 185 of 651 individuals (28.42%) were identified as having incident frailty according to Fried phenotype criteria. Individuals who experienced a CVD event at an older age, were pre-frail, and had polypharmacy at the time of an event were associated with higher odds of having incident Fried frailty (Supplemental Table S6). Similar associations were also observed in a sensitivity analysis that excluded participants with a second CVD event during the study period after their initial event (Supplemental Table S7). In additional analyses excluding participants who were already pre-frail before their CVD event, stroke survivors living in outer regional or remote areas had higher odds of developing Fried frailty (OR 4.29) (Supplemental Table S8) compared with those in major cities.

## Discussion

To our knowledge, this is the first study examining the risk of incident frailty after a CVD event among community-dwelling older people. We found that 333 individuals (45.12%) had an incident FI-defined frailty over an average of 2.6 years after a CVD event. Individuals who were pre-frail before the CVD event experienced notably higher odds of progressing to frailty afterwards. This association was consistently found across CVD subtypes, ie, MI and stroke. In addition, older age, being a woman, living in inner regional or outer regional/remote areas (vs metropolitan), and polypharmacy were associated with higher odds of experiencing incident FI-defined frailty following a CVD event. Our study found no strong evidence that the odds of developing incident frailty varied according to living status and SES.

Our prior work, which assessed the impact of a CVD event on frailty trajectory patterns as individuals age, provided robust evidence that the frailty burden not only worsens immediately after a CVD event, but also continues to increase over time.^26^ The present study builds on those findings by identifying factors associated with incident frailty after a CVD event. Together, these findings highlight the need to integrate frailty management into CVD treatment to delay the onset of frailty or mitigate frailty progression and minimise the burden of frailty-related complications in patients who have had a CVD event. Importantly, this study helps address a gap in knowledge around the early identification of those individuals at the highest risk of becoming frail after a CVD event, and thus it could inform targeted early interventions.

Our main finding was that individuals with pre-frailty status before a CVD event are at greater odds of developing frailty afterwards. The potential explanation could be the combined effects of CVD-induced physiologic stress on already weakened biological systems coupled with the consequences of post-CVD changes such as dependence on multiple medications (eg, polypharmacy), decreased physical mobility, and increased social and psychologic stress^35,36^ all contributing to the worsening progression into frailty. Furthermore, our finding aligns with prior understanding that pre-frailty, an intermediate state between robustness and frailty, is characterised by a decline in physiologic reserves and thus being more vulnerable to adverse health outcomes and less resilient to a major event such as CVD.^37^ Although our finding is not surprising, the present study contributes valuable insight to the clinical field, suggesting that pre-frail status could be considered an early marker of heightened risk for experiencing a worse prognosis after a CVD event and a critical target point for interventions aimed at reversing the frailty process or delaying its progression.

Our finding that residing in regional or remote areas is associated with increased odds of developing frailty after a CVD event is supported by previous studies that demonstrated geographic disparity in health outcomes, including mortality and well-being, among older people.^38,39^ Our study is the first to explore the impact of specific geographic location on frailty progression after CVD, providing new evidence regarding the risk of developing frailty in this context. Our findings could be attributed to health inequities in remote areas, such as longer travel times to access health care, challenges in receiving early revascularisation with thrombolysis or thrombectomy for acute stroke, limited availability of medical facilities or specialised cardiac and stroke rehabilitation programs, and poorer social support.^40,41^ These circumstances could make people from regional and remote areas more vulnerable to experiencing poorer health outcomes such as frailty development than their urban counterparts, particularly after a critical health event such as a stroke. Thus, the findings from our present study also highlight the importance of tailored health care strategies, including improved telehealth services and better access to rehabilitation and community-based social support,^42^ for CVD survivors in regional and remote areas.

### Clinical implications

Our findings provide clear evidence of higher odds of developing frailty among community-dwelling people after a CVD event. This highlights the need for health care providers to be aware of heightened frailty risk in people with a CVD event and the importance of frailty screening^43^ in this population to initiate timely interventions to reduce the burden of frailty-related complications and improve overall prognosis. In addition, identifying individuals who were pre-frail before a CVD event could be instrumental in helping clinicians pinpoint those at greatest risk of developing frailty, thereby allowing for proactive interventions. Tailored strategies for frailty management^10,42,44,45^ should be incorporated into CVD treatment and could include physical therapy, exercise programs, psychosocial and nutritional support, and regular follow-up to monitor frailty progression, particularly for CVD survivors who are of advanced age, females, experiencing polypharmacy, and residing in regional or remote areas.

### Strengths and limitations

This study has both strengths and limitations. A particular strength is its focus on the risk of developing frailty after a CVD event among community-dwelling individuals. This provides new insights into the field and makes our findings highly applicable for post-CVD frailty identification and intervention strategies in primary care. Furthermore, over an average of 8.3 years, ASPREE has followed participants without CVD for an incident CVD event, and conducted rigorous ascertainment and adjudication of CVD events rather than relying on administrative data sets.^23^ This enhances the study quality and the reliability of our findings. Moreover, ASPREE has collected extensive clinical and phenotypic data annually and continuously tracked frailty status over time. This data enables us to examine the association between pre-event frailty status (nonfrail vs pre-frail) and the odds of developing frailty after an event. This aspect is a particular strength of our study, making it unique compared with previous hospital-based studies that usually lack such information, ie, pre-event frailty status,^12^ demonstrating how pre-event frailty status affects the odds of developing frailty after a CVD event. In addition, although our average 2.6 years of follow-up for the risk of incident frailty after CVD events may seem relatively short, this time frame could be considered as practically relevant, because it allows health professionals to monitor health complications in the short term after a CVD event, thereby facilitating timely targeted intervention to improve overall prognosis. However, it is acknowledged that most of our study participants (88.8%) were identified as White Australians. This could limit the generalisability of our findings to other ethnoracial groups, highlighting an area for further study. In addition, it is well recognised that the specific type of CVD treatment, such as coronary artery bypass grafting, the placement of implantable cardiac defibrillators, and early revascularisation with thrombolysis or thrombectomy in acute stroke, can influence health outcomes, including frailty burden after a CVD event.^46^ Therefore, the lack of detailed CVD treatment information limits our study’s ability to account for these factors in the present analysis and should be acknowledged as a limitation of this study.

## Conclusion

This study, the first of its kind undertaken among community-dwelling older people, provides robust evidence that older age, being a woman, living in regional/remote areas, having polypharmacy, and pre-frail status markedly increased the odds of developing frailty after a CVD event. These findings should help clinicians to identify CVD patients most at risk of frailty and underscore the need for targeted interventions aimed at preventing frailty progression in vulnerable individuals. This proactive approach could enhance recovery outcomes and improve overall prognosis and well-being among people with a CVD event.

## Supplementary Material

Supplementary

To access the supplementary material accompanying this article, visit the online version of the *Canadian Journal of Cardiology* at www.onlinecjc.ca and at https://doi.org/10.1016/j.cjca.2025.06.003.

## Figures and Tables

**Central Illustration.**
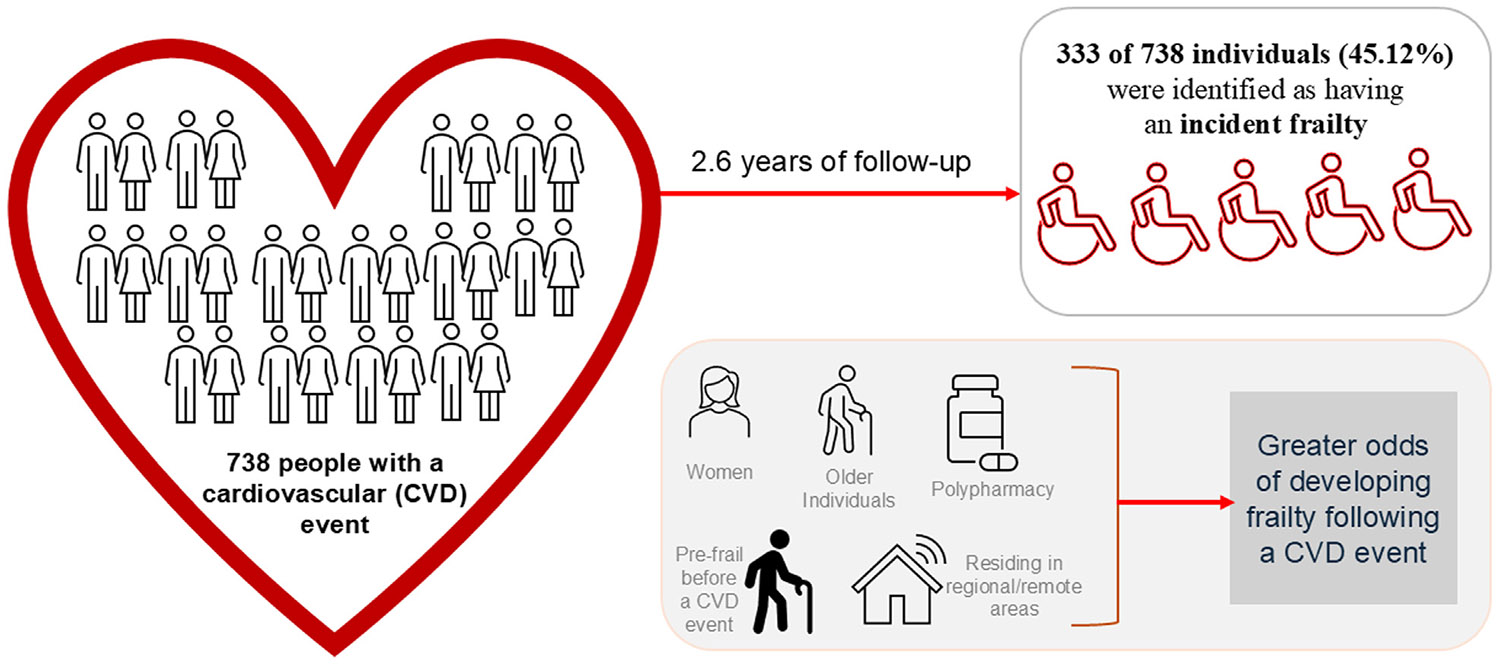
Frailty risk after a cardiovascular event in community-dwelling older people: Influence of sociodemographic, polypharmacy and pre-event frailty status. The illustration shows frailty risk after a cardiovascular disease (CVD) event among 1738 community-dwelling older people and summarizes key factors associated with higher odds of developing frailty following a CVD event

**Table 1.** Participants’ characteristics prior to a CVD event (n = 738)

Characteristics	Number (%)
Age, y, mean ≥ SD	79.3 ≥ 5.0
Sex	
Male	454 (61.5)
Female	284 (38.5)
Ethnoracial group	
White Australian	655 (88.8)
White American	46 (6.2)
Black/African American	16 (2.2)
Hispanic/Latino/Asiatic/others	21 (2.9)
Living status	
Living alone	262 (35.5)
Living with others	476 (64.5)
Area of residence*	
Major cities of Australia	322 (48.5)
Inner regional Australia	256 (38.6)
Outer regional or remote Australia	86 (13.0)
Socioeconomic status*	
Low	155 (23.3)
Middle	255 (38.4)
High	254 (38.3)
Frailty status before a CVD event	
Nonfrail	298 (40.4)
Pre-frail	440 (59.6)
Polypharmacy	
< 5 medications	396 (53.7)
≥ 5 medications	342 (46.3)

Values are n (%) unless otherwise specified.

CVD, cardiovascular disease.

* Information on the area of residence and socioeconomic status was available only for Australian participants (n = 664 participants).

**Table 2.** Associations between sociodemographic factors, polypharmacy, and pre-event frailty status and the odds of incident frailty after a CVD event (n = 738)

	OR	95% CI
Age	**1.04**	**1.01-1.08**
Sex		
Male	Ref.	
Female	**1.83**	**1.35-2.48**
Living status		
Living alone	Ref.	
Living with others	0.74	0.54-1.03
Area of residence		
Major cities of Australia	Ref.	
Inner regional Australia	**1.52**	**1.09-2.14**
Outer regional or remote Australia	1.12	0.69-1.83
Socioeconomic status		
Low	Ref .	
Middle	1.18	0.78-1.78
High	1.00	0.66-1.51
Frailty status before a CVD event		
Nonfrail	Ref.	
Pre-frail	**3.41**	**2.47-4.71**
Polypharmacy		
< 5 medications	Ref.	
≥ 5 medications	**1.37**	**1.02-1.84**

The table presents the results of logistic regression models adjusted for age and sex. Results in bold are significant (*P* < 0.05).

CI, confidence interval; CVD, cardiovascular disease; OR, odds ratio.

**Table 3.** Associations between sociodemographic factors, polypharmacy, and pre-event frailty status and the odds of incident frailty after myocardial infarction (MI) and stroke

	MI (n = 344)		Stroke (n = 301)	
	OR	95% CI	OR	95% CI
Age	**1.07**	**1.02-1.13**	1.04	1.00-1.10
Sex				
Male	Ref.		Ref.	
Female	**2.16**	**1.34-3.47**	1.48	0.93-2.35
Living status				
Living alone	Ref.		Ref.	
Living with others	0.69	0.42-1.14	0.90	0.54-1.49
Area of residence				
Major cities of Australia	Ref.		Ref.	
Inner regional Australia	1.28	0.78-2.09	**2.13**	**1.23-3.69**
Outer regional or remote Australia	0.79	0.36-1.75	**2.37**	**1.13-4.97**
Socioeconomic status				
Low	Ref.		Ref.	
Middle	0.74	0.40-1.36	1.60	0.84-3.05
High	1.21	0.66-2.20	0.75	0.39-1.41
Frailty status before a CVD event				
Nonfrail	Ref.		Ref.	
Pre-frail	**4.45**	**2.59-7.65**	**3.40**	**2.09-5.54**
Polypharmacy				
< 5 medications	Ref.		Ref.	
≥ 5 medications	1.10	0.70-1.73	1.37	0.87-2.18

A total of 134 (38.95%) of 344 individuals with MI were identified as having incident frailty according to FI; 147 (48.84%) of 301 individuals with stroke were identified as having incident frailty according to FI. The table presents the results of logistic regression models adjusted for age and sex. Results in bold are significant (*P* < 0.05).

CI, confidence interval; CVD, cardiovascular disease; OR, odds ratio.

## Data Availability

Data from the ASPREE study are available on reasonable request to qualified researchers, subject to approval of the analyses by the principal investigators and a standard data-sharing agreement. Details regarding requests to access the data will be available through the ASPREE Access Management Site (AMS): https://ams.aspree.org.
