# Supplementary material for: Frailty Risk After a Cardiovascular Event Among Community-Dwelling Older People: Influence of Sociodemographic, Polypharmacy, and Pre-event Frailty
Source: Can J Cardiol. Author manuscript; Available in PMC 2026 May 29. (PMC13221273; doi:10.1016/j.cjca.2025.06.003)
Supplement: Supplementary [file NIHMS2175695-supplement-Supplementary.pdf]

Supplemental Tables

Supplemental Table S1. Associations between sociodemographic factors, polypharmacy, pre-event frailty status and the odds of incident frailty after a CVD event, when excluding 44 participants who experienced a second CVD event during the study period after their initial event (N = 694)

	**OR**	**95% CI**
Age	**1.05**	**1.02 – 1.08**
Gender		
Men	Ref	
Women	**1.90**	**1.39 – 2.60**
Living status		
Living alone	Ref	
Living with others	0.74	0.53 – 1.04
Area of residence		
Major cities of Australia	Ref	
Inner regional Australia	**1.51**	**1.07 – 2.15**
Outer regional or remote Australia	1.15	0.69 – 1.90
Socioeconomic status		
Low	Ref	
Middle	1.22	0.80 – 1.86
High	0.93	0.61 – 1.43
Frailty status before a CVD event		
No frail	Ref	
Pre-frail	**3.72**	**2.65 – 5.21**
Polypharmacy		
<5 medications	Ref	
≥5 medications	**1.47**	**1.08 – 2.00**

CI, Confidence interval; CVD, Cardiovascular disease; OR, Odds ratio.

Note: 304 of 694 individuals with CVD (43.80%) were identified as having incident frailty. The table shows the results of logistic regression models adjusted for age and gender. Bold represents a significant result at p <0.05.

Supplemental Table S2 Associations between sociodemographic factors, polypharmacy, pre-event frailty status and the odds of incident frailty after a CVD event

	**CVD Adjusted model 1 N = 726**		**CVD Adjusted model 2 N = 652**	
	**OR**	**95% CI**	**OR**	**95% CI**
Age	1.04	1.00 – 1.07	1.04	1.00 – 1.08
Gender				
Men	Ref		Ref	
Women	**1.71**	**1.19 – 2.45**	**1.89**	**1.28 – 2.80**
Living status				
Living alone	Ref		Ref	
Living with others	0.81	0.57 – 1.14	0.87	0.60 – 1.26
Area of residence				
Major cities of Australia			Ref	
Inner regional Australia			**1.89**	**1.25 – 2.86**
Outer regional or remote Australia			1.33	0.73 – 2.42
Socioeconomic status				
Low			Ref	
Middle			1.07	0.68 – 1.70
High			1.33	0.78 – 2.24
Frailty status before a CVD event				
No frail	Ref		Ref	
Pre-frail	**3.48**	**2.41 – 5.02**	**3.48**	**2.35 – 5.15**
Polypharmacy				
<5 medications	Ref		Ref	
≥5 medications	1.19	0.84 – 1.68	1.22	0.85 – 1.76

CI, Confidence interval; CVD, Cardiovascular disease; OR, Odds ratio.

Adjusted model 1: Adjustment for age, gender, living status, ethno-racial group, smoking status, pre-event frailty status, hypertension, diabetes, dyslipidemia, obesity, depression, 3MS score, and eGFR_CKD score

Adjusted model 2: Additional adjustment for area of residence and SES

Bold represents a significant result at $p < 0.05$.

Supplemental Table S3. Associations between sociodemographic factors, polypharmacy, pre-event frailty status and the odds of incident frailty after MI

	**MI Adjusted model 1 N = 338**		**MI Adjusted model 2 N = 317**	
	**OR**	**95% CI**	**OR**	**95% CI**
Age	**1.08**	**1.02 – 1.14**	**1.07**	**1.00 – 1.13**
Gender				
Men	Ref		Ref	
Women	**2.00**	**1.12 – 3.60**	**2.18**	**1.17 – 4.06**
Living status				
Living alone	Ref		Ref	
Living with others	0.86	0.50 – 1.49	0.90	0.51 – 1.59
Area of residence				
Major cities of Australia			Ref	
Inner regional Australia			1.67	0.87 – 3.19
Outer regional or remote Australia			1.02	0.38 – 2.78
Socioeconomic status				
Low			Ref	
Middle			0.67	0.34 – 1.33
High			1.57	0.70 – 3.49
Frailty status before a CVD event				
No frail	Ref		Ref	
Pre-frail	**4.32**	**2.33 – 8.01**	**4.24**	**2.20 – 8.14**
Polypharmacy				
<5 medications	Ref		Ref	
≥5 medications	0.78	0.45 – 1.36	0.82	0.46 – 1.47

CI, Confidence interval; CVD, Cardiovascular disease; MI; Myocardial infarction; OR, Odds ratio.

Adjusted model 1: Adjustment for age, gender, living status, ethno-racial group, smoking status, pre-event frailty status, hypertension, diabetes, dyslipidemia, obesity, depression, 3MS score, and eGFR_CKD score

Adjusted model 2: Additional adjustment for area of residence and SES

Bold represents a significant result at $p < 0.05$.

Supplemental Table S4. Associations between sociodemographic factors, polypharmacy, pre-event frailty status and the odds of incident frailty after stroke

	**Stroke Adjusted model 1 N = 295**		**Stroke Adjusted model 2 N = 257**	
	**OR**	**95% CI**	**OR**	**95% CI**
Age	1.02	0.97 – 1.08	1.04	0.98 – 1.11
Gender				
Men	Ref		Ref	
Women	1.41	0.82 – 2.43	1.39	0.75 – 2.57
Living status				
Living alone	Ref		Ref	
Living with others	0.88	0.51 – 1.53	1.01	0.55 – 1.87
Area of residence				
Major cities of Australia			Ref	
Inner regional Australia			**3.33**	**1.64 – 6.77**
Outer regional or remote Australia			**2.98**	**1.17 – 7.57**
Socioeconomic status				
Low			Ref	
Middle			1.79	0.83 – 3.87
High			1.37	0.58 – 3.20
Frailty status before a CVD event				
No frail	Ref		Ref	
Pre-frail	**3.89**	**2.24 – 6.75**	**4.42**	**2.36 – 8.28**
Polypharmacy				
<5 medications	Ref		Ref	
$\geq$5 medications	1.30	0.75 – 2.24	1.42	0.77 – 2.63

CI, Confidence interval; CVD, Cardiovascular disease; OR, Odds ratio.

Adjusted model 1: Adjustment for age, gender, living status, ethno-racial group, smoking status, pre-event frailty status, hypertension, diabetes, dyslipidemia, obesity, depression, 3MS score, and eGFR_CKD score

Adjusted model 2: Additional adjustment for area of residence and SES

Bold represents a significant result at $p < 0.05$.

Supplemental Table S5. Associations between sociodemographic factors, and polypharmacy, and the odds of incident frailty after a CVD event, myocardial infarction (MI) and stroke, when additionally excluding participants who were already pre-frail prior to a CVD event

	**CVD (N = 298)**		**MI (N = 127)**		**Stroke (N = 137)**	
	**OR**	**95% CI**	**OR**	**95% CI**	**OR**	**95% CI**
Age	1.02	0.96 – 1.08	1.07	0.96 – 1.19	1.00	0.92 – 1.09
Gender						
Men	Ref		Ref		Ref	
Women	**2.16**	**1.27 – 3.70**	1.92	0.66 – 5.59	**2.09**	**1.01 – 4.33**
Living status						
Living alone	Ref		Ref		Ref	
Living with others	0.82	0.45 – 1.48	1.93	0.47 – 7.87	0.66	0.30 – 1.45
Area of residence						
Major cities of Australia	Ref		Ref		Ref	
Inner regional Australia	1.46	0.81 – 2.63	0.96	0.33 – 2.75	1.87	0.79 – 4.43
Outer regional or remote Australia	1.28	0.51 – 3.20	1.31	0.24 – 7.20	1.45	0.41 – 5.08
Socioeconomic status						
Low	Ref		Ref		Ref	
Middle	1.27	0.59 – 2.73	0.80	0.22 – 2.91	1.49	0.52 – 4.32
High	1.18	0.56 – 2.48	0.96	0.28 – 3.32	1.04	0.37 – 2.95
Polypharmacy						
<5 medications	Ref		Ref		Ref	
≥5 medications	1.07	0.62 – 1.87	1.37	0.52 – 3.57	1.08	0.49 – 2.37

CI, Confidence interval; CVD, Cardiovascular disease; MI; Myocardial infarction; OR, Odds ratio. **Note:** 80 of 298 individuals with CVD (26.85%) were identified as having incident FI-defined frailty. 22 of 127 individuals with MI (17.32%) were identified as having incident FI-defined frailty. 44 of 137 individuals with stroke (32.12%) were identified as having incident FI-defined frailty. The table shows the results of logistic regression models adjusted for age and gender. Bold represents a significant result at p <0.05.

Supplemental Table S6. Associations between sociodemographic factors, polypharmacy, pre-event frailty status and the odds of incident Fried frailty after a CVD event, myocardial infarction (MI) and stroke

	**CVD (N = 651)**		**MI (N = 301)**		**Stroke (N = 255)**	
	**OR**	**95% CI**	**OR**	**95% CI**	**OR**	**95% CI**
Age	**1.07**	**1.03 – 1.10**	1.06	1.00 – 1.13	1.06	1.00 – 1.12
Gender						
Men	Ref		Ref		Ref	
Women	0.87	0.61 – 1.24	0.79	0.44 – 1.41	0.96	0.56 – 1.64
Living status						
Living alone	Ref		Ref		Ref	
Living with others	0.75	0.52 – 1.10	0.62	0.34 – 1.13	0.90	0.51 – 1.61
Area of residence						
Major cities of Australia	Ref		Ref		Ref	
Inner regional Australia	1.30	0.88 – 1.92	1.27	0.70 – 2.31	1.54	0.83 – 2.86
Outer regional or remote Australia	1.23	0.69 – 2.22	0.96	0.38 – 2.44	1.91	0.78 – 4.65
Socioeconomic status						
Low	Ref		Ref		Ref	
Middle	1.01	0.62 – 1.64	1.29	0.60 – 2.75	1.08	0.51 – 2.27
High	0.79	0.49 – 1.28	1.07	0.50 – 2.29	0.66	0.31 – 1.39
Frailty status before a CVD event						
No frail	Ref		Ref		Ref	
Pre-frail	**2.69**	**1.84 – 3.93**	**4.02**	**2.16 – 7.49**	**2.02**	**1.14 – 3.59**
Polypharmacy						
<5 medications	Ref		Ref		Ref	
≥5 medications	**1.77**	**1.24 – 2.52**	1.58	0.91 – 2.75	**1.99**	**1.15 – 3.44**

CI, Confidence interval; CVD, Cardiovascular disease; MI; Myocardial infarction; OR, Odds ratio. **Note:** 70 of 301 individuals with MI (23.26%) were identified as having incident Fried frailty. 80 of 255 individuals with stroke (31.37%) were identified as having incident Fried frailty. The table shows the results of logistic regression models adjusted for age and gender. Bold represents a significant result at $p < 0.05$.

Supplemental Table S7. Associations between sociodemographic factors, polypharmacy, pre-event frailty status and the odds of incident Fried frailty after a CVD event, when excluding 29 participants who experienced a second CVD event during the study period after their initial event (N = 622)

	OR	95% CI
Age	**1.07**	**1.03 – 1.11**
Gender		
Men	Ref	
Women	0.90	0.62 – 1.29
Living status		
Living alone	Ref	
Living with others	0.79	0.53 – 1.15
Area of residence		
Major cities of Australia	Ref	
Inner regional Australia	1.37	0.91 – 2.05
Outer regional or remote Australia	1.25	0.68 – 2.30
Socioeconomic status		
Low	Ref	
Middle	0.96	0.59 – 1.58
High	0.70	0.42 – 1.15
Frailty status before a CVD event		
No frail	Ref	
Pre-frail	**2.94**	**1.97 – 4.37**
Polypharmacy		
<5 medications	Ref	
≥5 medications	**1.78**	**1.23 – 2.58**

CI, Confidence interval; CVD, Cardiovascular disease; OR, Odds ratio.

Note: 171 of 622 individuals with CVD (27.49%) were identified as having incident Fried frailty. The table shows the results of logistic regression models adjusted for age and gender. Bold represents a significant result at $p < 0.05$.

Supplemental Table S8. Associations between sociodemographic factors, and polypharmacy, and the odds of incident Fried frailty after a CVD event, myocardial infarction (MI) and stroke, when additionally excluding participants who were already pre-frail prior to a CVD event

	**CVD (N = 288)**		**MI (N = 144)**		**Stroke (N = 112)**	
	**OR**	**95% CI**	**OR**	**95% CI**	**OR**	**95% CI**
Age	1.05	0.98 – 1.13	1.00	0.88 – 1.13	1.05	0.95 – 1.16
Gender						
Men	Ref		Ref		Ref	
Women	0.97	0.51 – 1.84	1.62	0.53 – 4.97	0.96	0.39 – 2.33
Living status						
Living alone	Ref		Ref		Ref	
Living with others	0.52	0.26 – 1.03	0.50	0.15 – 1.64	0.52	0.20 – 1.38
Area of residence						
Major cities of Australia	Ref		Ref		Ref	
Inner regional Australia	1.14	0.57 – 2.30	1.69	0.56 – 5.08	1.18	0.39 – 3.62
Outer regional or remote Australia	1.86	0.75 – 4.59	0.63	0.07 – 5.60	**4.29**	**1.22 – 15.08**
Socioeconomic status						
Low	Ref		Ref		Ref	
Middle	1.12	0.49 – 2.54	1.82	0.45 – 7.45	0.84	0.26 – 2.71
High	0.71	0.30 – 1.67	1.14	0.25 – 5.14	0.42	0.12 – 1.43
Polypharmacy						
<5 medications	Ref		Ref		Ref	
≥5 medications	1.31	0.70 – 2.44	1.32	0.45 – 3.88	0.93	0.37 – 2.36

CI, Confidence interval; CVD, Cardiovascular disease; MI; Myocardial infarction; OR, Odds ratio. **Note:** 49 of 288 individuals with CVD (17.01%) were identified as having incident Fried frailty. 16 of 144 individuals with MI (11.11%) were identified as having incident Fried frailty. 25 of 112 individuals with stroke (22.32%) were identified as having incident Fried frailty. The table shows the results of logistic regression models adjusted for age and gender. Bold represents a significant result at $p < 0.05$.
